# Sex-Related Differences in Neurosensory Alterations Following Blunt Head Injury

**DOI:** 10.3389/fneur.2020.01051

**Published:** 2020-09-15

**Authors:** Angela Lumba-Brown, Kian Niknam, Jordan Cornwell, Courtney Meyer, Jamshid Ghajar

**Affiliations:** ^1^Department of Emergency Medicine, Stanford University, Palo Alto, CA, United States; ^2^University of California, San Francisco Medical School, San Francisco, CA, United States; ^3^Athletic Training, Department of Sports Medicine, Stanford University, Palo Alto, CA, United States; ^4^Department of Neurosurgery, Brain Performance Center, Stanford University, Palo Alto, CA, United States

**Keywords:** concussion, mild traumatic brain injuries, vestibular, oculomotor, subtypes, neurosensory alterations, sports related concussion

## Abstract

**Background:** There is heterogeneity in neurosensory alterations following mild traumatic brain injury. Commonly assessed neurosensory symptoms following head injury include symptom reports and measures of oculomotor impairment, auditory changes, and vestibular impairment.

**Hypothesis/Purpose:** Neurosensory alterations are prevalent acutely following mild traumatic brain injury secondary to blunt head trauma during collegiate varsity sports and may vary by sex and sport.

**Study Design:** Retrospective study of a large collegiate athletic database.

**Methods:** Analyses were performed using an established single University dataset of 177 male and female collegiate varsity athletes who were diagnosed with concussion/mild traumatic brain injury between September 2013 and October 2019. Descriptive and comparative analyses were performed on individual and grouped acute concussion assessments pertaining to neurosensory alterations obtained within 72 h of injury using components of the Sports Concussion Assessment Tool Version 5 and Vestibular/Ocular-Motor Screening.

**Results:** Females had significantly more abnormal smooth pursuit (*p*-value: 0.045), convergence (*p*-value: 0.031), and visual motion sensitivity tests results (*p*-value: 0.023) than males. There were no differences in neurosensory alterations when grouped by *overall* auditory, vestibular, or oculomotor impairments. The majority of sports-related concussions occurred during football (50, 28.25%), wrestling (21, 11.86%), water polo (15, 8.47%), and basketball (14, 7.91%). Abnormal vestibular assessments were high in these top four sports categories, but statistically significant differences in overall auditory, vestibular, or oculomotor impairments were not reached by individual sport. However, water polo players had higher abnormal individual assessments related to balance reports on the sideline (60.00%, *p*-value: 0.045) and in the clinic setting (57.14%, *p*-value: 0.038) as compared to all other sports.

**Conclusion:** While neurosensory alterations are prevalent in both male and female athletes acutely post-concussion, females have a higher incidence of abnormalities in smooth pursuit, convergence, and visual motion sensitivity and may benefit from early rehabilitation.

## Introduction

Neurosensory alterations following blunt head trauma can be debilitating because sensory input informs how we interact with the world. Heterogeneity in neurosensory alterations following mild traumatic brain injury (TBI), including concussion, may be related to premorbid conditions, medications, injury-related factors, and other modifying factors such as co-existing injuries or intoxication (1, 2). Some neurosensory-related symptoms are assessed in common post-concussion symptom scales and measures of vestibulo-oculomotor impairment following mild TBI in children and adults (3–5).

Neurosensory alterations are examined to various degrees in a variety of concussion assessments employed acutely, throughout recovery, and in the long-term setting. For example, the assessment of symptom burden and symptom provocation following vestibulo-oculomotor assessment has been reported in pediatric athletes in the acute setting (5, 6). Visual processing impairments are identifiable in adults following childhood concussion, upon long-term assessment (7). Though outcomes of tests used for concussion assessment have been reported at varying time-points following injury, there is limited data reporting thematically-grouped neurosensory alterations using subjective and objective measures following acute blunt head injury resulting in sports-related concussion in men and women.

Understanding the comparative prevalence of neurosensory alterations in physically fit individuals in the acute setting is particularly important to inform the range of acute impairments that may be seen in soldiers injured in austere environments, where traditional concussion assessment may not be feasible. We hypothesized that neurosensory alterations are acutely prevalent in physically fit, varsity collegiate athletes following on-field, blunt head injury resulting in sports-related concussion and that these symptoms and impairments would be higher in sports with more frequent person-to-person blunt impacts such as football and wrestling. This study presents the immediate, on-scene reports of neurosensory symptoms in varsity collegiate athletes, as well as reports from the initial concussion clinic visit within 72-h of injury.

## Methods

This study was a retrospective review of an existing sports concussion database of collegiate varsity athletes sustaining concussion at a single university. All athletes are evaluated by a concussion specialist (a neurosurgeon or sports-medicine physician) and diagnosed with concussion in accordance with the 5th Consensus Statement on Concussion in Sport recommendations upon their first clinic visit within 72-h of injury (8). Athletes completed post-concussion symptom assessments on the sideline, immediately following injury, as well as at the first concussion clinic visit. Clinic assessments included the complete Sports Concussion Assessment Tool Version 5 (SCAT5), Vestibular/Ocular-Motor Screening (VOMS), and eye-tracking utilizing virtual reality goggles. Athletes sustaining concussion from 2013 to 2019 were included in this analysis.

### Measures

The SCAT5 is a concussion assessment tool that includes a post-concussive symptom evaluation, memory and cognitive screen, Glasgow Coma Scale score, and balance assessment (9). The symptom evaluation employs a rating scale of 22 symptoms with severity ratings from 0 (no symptom) to 6 (maximum symptom). Balance is assessed at the clinic visit using the Balance Error Scoring System (BESS) and on the sideline using the modified BESS.

The Vestibular Ocular Motor Screening (VOMS) measures symptom provocation while testing the ability of a patient to complete a set of balance, vision, and movement-integrated tasks (10). In our cohort, this assessment was completed by physical therapists in the initial concussion clinic visit. Patients were prompted to rate their symptom levels on a scale from 0 to 10 before testing began and following each task. The 4 patient-reported symptoms queried are “headache,” “dizziness,” “nausea,” and “fogginess.” Our cohort performed 7 tests related to the VOMS: smooth pursuit, horizontal and vertical saccades, convergence, horizontal and vertical VOR, and visual motion sensitivity.

Our institution's internal review board approved the population of this research database (IRB-47124) and de-identified data analysis was adjudicated as exempt.

### Dichotomization and Statistical Analysis

Continuous scores from the SCAT5 and individual tests comprising the VOMS were dichotomized into two groups: normal and abnormal. Modified BESS and sideline symptoms were taken from the SCAT5, whereas clinic BESS, clinic symptoms, and VOMS were taken from the physical therapy screening performed within 72 h of injury at the initial concussion clinic visit with the diagnosing neurosurgeon or sports medicine physician. Sideline modified BESS and clinic BESS scores of ≥5 and ≥15, respectively, were designated as abnormal (11). The presence of a symptom, of any severity, at sideline or clinic was labeled as abnormal. For each VOMS assessment, any single symptom with a severity ≥2 or a near point convergence of ≥5 cm was marked as abnormal (10).

A novel categorization system was used to dichotomize the BESS, VOMS, and reported symptoms as normal or abnormal based on auditory, vestibular, or oculomotor impairments. Abnormal vestibular assessments included any abnormal scoring on components of the VOMS, BESS, or symptoms of “dizziness” and/or “balance problems.” Abnormal oculomotor assessments included abnormal scoring on components of the VOMS or symptoms including “vision changes,” “light sensitivity,” or “blurred vision.” Abnormal auditory reports were designated as the presence of “noise sensitivity” or “ringing in ears.”

Descriptive statistics were calculated for the subjects' demographics and scores on concussion assessments. Dichotomized variables were analyzed using chi squared or Fisher's exact test, as appropriate. Logistic regression was used to assess effect size, and Levene's test was used to asses equalities in variance between males and females with regards to outcomes. Analyses were run using Stata 14/SE for Windows (StataCorp, LP College Station, TX).

## Results

We identified 177 varsity collegiate athletes diagnosed with sports-related concussion/mild TBI following blunt head injury from 2013 to 2019. Within this population, 103 (58.19%) were male, and 74 (41.81%) were female, as reported in Table 1. The subjects participated in a variety of sports, with the majority of sports-related concussion occurring from football (50, 28.25%), wrestling (21, 11.86%), water polo (15, 8.47%), and basketball (14, 7.91%).

**Table 1:** Demographics and subject characteristics.

**Characteristic**	***N* (177)**	**Percentage**
**Sex**	**177**	
Male	103	58.19%
Female	74	41.81%
**Sports**	**177**	
Baseball	4	2.26%
Basketball	14	7.91%
Beach volleyball	6	3.39%
Fencing	4	2.26%
Field hockey	4	2.26%
Football	50	28.25%
Golf	1	0.56%
Gymnastics	6	3.39%
Lacrosse	7	3.95%
Rowing	8	4.52%
Sailing	7	3.95%
Soccer	8	4.52%
Softball	5	2.82%
Swimming and diving	7	3.95%
Synchronized swimming	1	0.56%
Track and field	1	0.56%
Volleyball	8	4.52%
Water polo	15	8.47%
Wrestling	21	11.86%

*The bolded numbers represent total number of subjects related to that column*.

Sex-based differences in specific portions of the VOMS were identified. Females had consistently more abnormal VOMS measurements with statistical significance being reached for smooth pursuit (*p*-value: 0.045), convergence (*p*-value: 0.031), and visual motion sensitivity tests (*p*-value: 0.023), as depicted in Table 2. Though males consistently had higher rates of light sensitivity and females had higher rates of noise sensitivity, these differences were not statistically significant, as reported in Tables 2, 3. No significant differences were found in *grouped* vestibular, oculomotor, or auditory assessments between males and females, described in Table 4.

**Table 2:** Vestibular and oculomotor impairment report by sex.

**Impairment report**	**Male Abnormal**	**Female Abnormal**			***p*-value**
**Vision changes**					
Sideline “blurred vision”	33	39.47%	18	32.93%	0.447
Clinic “vision change”	1	0.96%	4	5.34%	0.162
**Light sensitivity**					
Sideline	36	43.08%	31	56.96%	0.108
Clinic	10	9.62%	10	13.36%	0.430
**VOMS (clinic)**					
Smooth pursuit	58	67.98%	49	82.81%	0.045
Horizontal saccades	59	69.16%	50	83.10%	0.056
Vertical saccades	58	67.98%	47	80.77%	0.089
Convergence	64	75.08%	52	89.50%	0.031
Horizontal VOR	63	73.03%	49	84.26%	0.112
Vertical VOR	57	67.60%	46	81.88%	0.060
Visual motion sensitivity	55	70.25%	48	87.07%	0.023
**BESS**					
Sideline	34	40.67%	22	41.06%	0.950
Clinic	22	23.46%	26	35.79%	0.081
**Dizziness**					
Sideline	48	57.54%	32	58.82%	0.868
Clinic	35	33.76%	29	38.87%	0.477
**Balance impairment**					
Sideline	27	32.66%	24	43.99%	0.175
Clinic	2	1.92%	0	0.00%	0.511

**Table 3:** Auditory impairment report by sex.

**Hearing report**	**Male Abnormal**	**Female Abnormal**			***p*-value**
**“Noise sensitivity”**					
Sideline	33	39.47%	23	42.14%	0.742
Clinic	7	6.74%	8	10.68%	0.344
**Clinic “ringing in ears”**	1	0.96%	0	0.00%	1.000

**Table 4:** Neurosensory reports by sex.

**Neurosensory measurement**	**Male**	**Percentage**	**Female**	**Percentage**	***p*-value**
**Vestibular**	**103**		**74**		0.190
Normal	12	11.65%	4	5.41%	
Abnormal	91	88.35%	70	94.59%	
**Oculomotor**	**103**		**74**		0.258
Normal	19	18.45%	9	12.16%	
Abnormal	84	81.55%	65	87.84%	
**Auditory**	**103**		**74**		0.769
Normal	66	64.08%	49	66.22%	
Abnormal	37	35.92%	25	33.78%	

*The bolded numbers represent total number of subjects related to that column*.

Logistic regression was used to assess neurosensory alteration differences by sex. Male athletes had 0.43 times the odds of vestibular impairment (95%CI: 0.13–1.40, *p*-value: 0.163) and 0.61 times the odds of oculomotor impairment (95%CI: 0.26–1.44, *p*-value: 0.261) relative to females. However, males had 1.10 times the odds of having hearing issues following a blunt head injury relative to females (95%CI: 0.59–2.06, *p*-value: 0.769).

Levene's test was used to assess for variance equality between groups and their outcomes. We found no significant differences in variances between sex and vestibular (*p*-value: 0.155), oculomotor (*p*-value: 0.261), and hearing (*p*-value: 0.770) impairments.

When comparing overall and grouped assessments of vestibular, oculomotor, or auditory impairments, football players had higher rates of abnormal assessments, however, statistical significance was not reached, as reported in Table 5. Among the sports with the highest internal concussion rates (football, wrestling, basketball, and water polo), water polo players had significantly higher rates of abnormal BESS assessments (57.14%, *p*-value: 0.038) and reports of balance problems (60.00%, *p*-value: 0.045), in the clinic and sideline settings, respectively, as reported in Table 6. Abnormal VOMS and other oculomotor assessments were common in the top four sports, but statistically significant differences were not reached. Overall, abnormal vestibular and oculomotor assessments were more common than reports of abnormal auditory symptoms.

**Table 5:** Neurosensory alterations by sport.

	**Abnormal vestibular**		***p*-value**	**Abnormal oculomotor**		***p*-value**	**Abnormal auditory**		***p*-value**
**Sport categorized**	**161**		0.152	**149**		0.196	**62**		0.801
Football	42	26.09%		39	26.17%		19	30.65%	
Wrestling	18	11.18%		16	10.74%		7	11.29%	
Water polo	15	9.32%		15	10.07%		3	4.84%	
Basketball	13	8.07%		12	8.05%		5	8.06%	
**Top 4 sports combined**	**88**		0.424	**99**		0.186	**39**		0.665
**Other sports**	**73**		0.527	**17**		0.948	**5**		0.267

*The bolded numbers represent total number of subjects related to that column*.

**Table 6:** Vestibular impairment reports by sport.

**Sport**	**Football Abnormal**		**Wrestling Abnormal**		**Water Polo Abnormal**		**Basketball Abnormal**		**Other Sport Abnormal**		***p*-value 1**	***p*-value 2**
**VOMS**												
Smooth pursuit	28	66.67%	9	75.00%	12	85.71%	9	64.29%	49	79.03%	0.448	0.524
Horizontal saccades	28	66.67%	8	66.67%	12	85.71%	9	64.29%	52	82.54%	0.207	0.548
Vertical saccades	28	66.67%	8	66.67%	12	85.71%	8	61.54%	49	79.03%	0.351	0.524
Convergence	30	71.43%	10	83.33%	13	92.86%	11	84.62%	52	83.87%	0.439	0.374
Horizontal VOR	29	69.05%	10	76.92%	11	84.62%	10	71.43%	52	83.87%	0.404	0.786
Vertical VOR	27	64.29%	9	75.00%	10	83.33%	9	69.23%	48	78.69%	0.514	0.692
Visual motion sensitivity	27	69.23%	8	72.73%	10	83.33%	8	72.73%	50	83.33%	0.490	0.850
**BESS**												
Sideline	18	43.90%	7	38.89%	2	22.22%	3	33.33%	26	44.07%	0.797	0.708
Clinic	10	22.73%	2	11.76%	8	57.14%	3	21.43%	25	32.89%	0.058	0.038
**“Dizziness”**												
Sideline	21	52.50%	13	68.42%	6	60.00%	4	44.44%	36	61.02%	0.691	0.584
Clinic	14	28.00%	10	47.62%	6	40.00%	6	42.86%	28	36.36%	0.557	0.390
**“Balance problem”**												
Sideline	11	28.21%	10	52.63%	6	60.00%	1	11.11%	23	38.98%	0.089	0.045
Clinic	2	4.00%	0	0.00%	0	0.00%	0	0.00%	0	0.00%	0.461	1.000

## Discussion

This study demonstrated that females have higher rates of abnormal outcomes on specific measures of the VOMS (smooth pursuit, convergence, and visual motion sensitivity tests) as compared to males, which has been reported previously (12). The pathophysiology of this finding has yet to be determined. Females appear to be more susceptible to certain neurosensory alterations related to oculomotor control and motion perception, representing a potential injury vulnerability. Clinical assessment in the acute post-injury period is warranted to allow for the potential benefit of early rehabilitation. There is no difference in the *grouped* neurosensory alterations by sex, underscoring the importance of considering all outcomes derived from larger concussion assessment batteries.

The vast majority of concussions included in this dataset were sustained by subjects while playing football, wrestling, water polo, and basketball, which is consistent with previous reports of concussion incidence in sport. These sports also had the highest incidence of repeat concussions, though statistical significance when grouped by neurosensory alterations was not reached. This study reported greater neurosensory alterations in football, wrestling, water polo, and basketball compared to other sports present in our dataset including baseball, beach volleyball, fencing, field hockey, golf, gymnastics, lacrosse, sailing, softball, swimming and diving, synchronized swimming, track and field, rowing, soccer, and volleyball. Statistically significant differences in grouped auditory, vestibular, or oculomotor impairments were not reached by individual sport suggesting that mechanisms of injury in relation to a specific sport may not affect neurosensory alteration patterns. When examining individual outcomes by sport, water polo players had relatively more abnormal balance reports than other sports. Differences in balance assessments and training have been queried in previous literature, including comparing land to water-sports, but the significance is unclear (13, 14).

The strengths of this research are that 100% of subjects had complete reports of pre-defined neurosensory alterations pertaining to groupings of oculomotor, vestibular, and auditory symptoms and impairments at sideline and initial concussion clinic assessment. Much data has been reported on neurosensory alterations in sub-acute and chronic settings, especially pertaining to children (3–5, 7); however, this work represents the first study to report and compare grouped neurosensory outcomes in the immediate-acute time frame in highly physically fit collegiate athletes, by sex and sport. It also supports recent work identifying sex-based differences in vestibular-oculomotor assessments.

This study was limited by a small number of subjects populating individual “other sports” which included: baseball, beach volleyball, fencing, field hockey, golf, gymnastics, lacrosse, sailing, softball, swimming and diving, synchronized swimming, track and field, rowing, soccer, and volleyball. Identifying specific differences in neurosensory alterations in association with these sports requires a larger sample size pertaining to these sports. This study was also limited by the examination of inherently subjective and semi-objective measures, such as balance assessments and symptom scale reports, which may not sufficiently reflect the scope of symptomatology or impairment (14–16). Objective evaluation of neurosensory alterations, such as pin-prick testing, hearing examinations, and Snellen's chart for vision testing, were not obtained in this sample, limiting further interpretation of more objective impairments.

Concussion causes immediate neurosensory alterations, most commonly represented by vestibular and oculomotor symptoms and impairments, followed by auditory symptoms. While concussion recovery is traditionally defined by the resolution of post-concussive symptoms, bridging the gap between symptomatology and objective measures is critical to understanding the pathophysiology of injury and its recovery (16). Future research should aim to elucidate the pathophysiology of sex-based differences in order to address more personalized care including injury prevention strategies.

As our study demonstrated, important impairments may be determined from specific tests within larger, commonly used concussion assessments. In order to advance the field and allow for continued scientific hypotheses, the outcomes of specific tests within common concussion assessments should be analyzed for secondary outcomes, such as sex-based differences. Additionally, further research is needed to understand how targeted treatments may address these complaints early on, improving symptoms and quality of life in the acute setting, as well as shortening recovery trajectories in both females and males. Finally, research identifying neurosensory alterations contributing to common concussion subtypes, considering the presence or absence of cervical strain as an associated condition, will further inform overlap between physiologic systems and direct targeted treatments (1, 2).

## Data Availability Statement

The data analyzed in this study was subject to the following licenses/restrictions: the dataset has not been approved by Stanford University for dissemination. Requests to access these datasets should be directed to falene@stanford.edu.

## Ethics Statement

The studies involving human participants were reviewed and approved by Stanford University Internal Review Board. Written informed consent for participation was not required for this study in accordance with the national legislation and the institutional requirements.

## Author Contributions

AL-B, JC, and JG designed and conducted the study. AL-B, CM, and KN authored the manuscript. All authors contributed to major edits and revisions, and agree to be accountable for the content of the work.

## Conflict of Interest

The authors declare that the research was conducted in the absence of any commercial or financial relationships that could be construed as a potential conflict of interest.
